# Sexual Function and Libido Loss in Female Climbers—A Cross-Sectional Study

**DOI:** 10.3390/sports14060242

**Published:** 2026-06-11

**Authors:** Sabrina Vollrath, Anne-Sophie Koller, Norman Bitterlich, Ana Buadze, Alexandra S. Kohl Schwartz, Petra Stute, Anthony C. Hackney, Nathalie Werth

**Affiliations:** 1 Department of Obstetrics and Gynecology, Inselspital, University of Bern, 3010 Bern, Switzerland; 2 Section Performance Sport, Swiss Federal Institute of Sport—BASPO, 2532 Magglingen, Switzerland; 3 University of Bern, 3010 Bern, Switzerland; 4 Freelance Statistician “Analysing, Consulting, Training”, 09114 Chemnitz, Germany; 5 Department of Adult Psychiatry and Psychotherapy, University Hospital of Psychiatry Zurich, University of Zurich, 8008 Zurich, Switzerland; 6 Department of Obstetrics and Gynecology, Division of Reproductive Medicine and Gynecological Endocrinology, Universitary Hospital Lucerne, 6004 Lucerne, Switzerland; 7 Department of Nutrition, University of North Carolina, Chapell Hill, NC 27514, USA; 8 OMVIA AG, Fertility and Hormone Clinic, 6300 Zug, Switzerland

**Keywords:** libido, reproductive health, relative energy deficiency in sports, climbing, low energy availability

## Abstract

**Aim:** Increasing female participation in elite sports has highlighted the need to better understand how intensive training affects reproductive health and sexual function. The aim of this cross-sectional study was to assess the prevalence of libido loss and sexual dysfunction in female climbers and to explore potential associations with low energy availability within the “relative energy deficiency in sports” framework. **Methods:** This is a cross-sectional multinational cohort study of female climbers as well as non-athletic controls from Switzerland, Germany, Austria, and Italy, to investigate female sexual function in athletes compared to a non-athletic control group from the general population through specific questionnaires, e.g., eating disorder screening (EDE-QS), sexual function (FSFI-d), low energy availability (LEAF-Q), and estrogen deficiency symptoms (MRS-II). A nonparametric procedure was used to check whether distribution differences between the groups were detectable. Where distributional differences were statistically detectable, selected parameters were considered as covariates in an analysis of covariance (ANCOVA). This has been carried out with the following covariates: LEAF- Q, MRS-II-score, age, BMI, and subjective satisfaction. Only participants without the signs of an eating disorder (normal EDE-QS scores) were included in this study. **Results:** A total of 173 women were included (elite: n = 31, amateur: n = 55, controls: n = 87). No significant differences in overall sexual function (FSFI-d total score) (*p* = 0.518) and libido (*p* = 0.610) were observed between groups in unadjusted analyses. However, after adjustment for relevant covariates, including MRS-II score and subjective sexual satisfaction, elite climbers demonstrated significantly lower FSFI-d scores compared to controls (*p* = 0.018). Notably, elite climbers reported higher subjective sexual satisfaction than controls (*p* = 0.002). **Conclusions:** While overall sexual function did not differ in unadjusted analyses, adjusted comparisons suggest that elite climbers may experience subtle differences in sexual function.

## 1. Introduction

Sexual function, including sexual desire, is a key component of physical, psychological, and reproductive health and an important determinant of quality of life and overall well-being [1,2]. According to a recent cross-sectional study in Germany, the prevalence of impaired libido in the general female population is 19.2% for the age group 18–25 years and 32% for the age group 26–35 years [3]. While regular physical activity in the general population is associated with improved sexual function [4,5], increasing evidence, particularly in male athletes suggests that extreme training loads, insufficient recovery, and inadequate energy intake or availability may adversely affect reproductive and especially sexual health in high-performance athletes [6,7]. One of the topics that has received little attention is the effect of intensive training on libido or sexual function in female athletes.

Evidence on sexual dysfunction in female athletes is limited, with most available data derived from studies in female cyclists [8,9]. In this context, reduced sexual function is predominantly investigated in relation to biomechanical factors (i.e., saddle design and riding position). These factors may directly affect genital tissues and pelvic floor structures, leading to pain, numbness, and pelvic floor symptoms, rather than reflecting systemic alterations of endocrine or reproductive function.

In contrast to biomechanical mechanisms and pelvic floor-related symptoms, low energy availability (LEA) represents a systemic condition affecting sexual and reproductive health in female athletes. The International Olympic Committee (IOC) consensus statement on relative energy deficiency in sports (REDs) describes impaired physiological function as a result of inadequate energy availability, affecting multiple systems, including metabolic, immunological, gastrointestinal, psychological, hematological, cardiovascular, and reproductive endocrine function via the central suppression of the hypothalamic–pituitary–gonadal axis in both female and male athletes [6,10]. The risk of LEA is particularly high in sports where athletes require low body weight and body fat to confer a performance advantage (i.e., so-called ‘lean sports’) [11]. As the direct assessment of energy availability is difficult in clinical and field settings, secondary indicators are commonly used for REDs screening. In female athletes, a key screening tool for REDs is the Low Energy Availability in Females Questionnaire (LEAF-Q), which incorporates menstrual, gastrointestinal, and injury-related symptoms [5]. In male athletes, reduced libido [6,12,13] is a well-described clinical feature of LEA or REDs in the context of high training volumes and is described as the exercise-hypogonadal male condition (EHMC) [7,14,15,16], which may be accompanied by reduced testosterone concentrations [12,17], and alterations of reproductive function in men [18]. In women, LEA is likewise associated with hypothalamic suppression, menstrual dysfunction, and hypoestrogenism; however, sexual function and sexual desire have rarely been assessed directly, and data are lacking for sports beyond cycling, particularly in female athletes exposed to high training loads or participating in lean sports with an increased risk of REDs.

Climbing represents a relevant model to address this gap as it is a sport with high physical demands and weight-dependent performance with potential risk for LEA. To date, no studies have specifically examined sexual function and libido in female climbers. We hypothesised that elite female climbers would demonstrate reduced sexual function and lower libido compared to controls, potentially associated with indicators of low energy availability within the REDs framework. Therefore, the aim of this cross-sectional study was to assess sexual function and libido in female climbers participating in a lean sport and to explore their association with indirect markers of low energy availability and REDs, including the LEAF-Q score [19], menstrual function [6], BMI [6], and estrogen deficiency-related symptoms [20].

## 2. Methods

### 2.1. Sample and Procedure

This is a cross-sectional multinational cohort study conducted in female climbers and non-athletic controls from German-speaking countries, including Switzerland, Germany, Austria, and the South Tyrol region of Italy. All participants were aged 16–35 years. Female amateur and elite athletes were eligible for inclusion in the examination group. Elite climbers were defined as athletes who are members of a national or regional team and who compete in national or international events. Amateur climbers were defined as individuals who train approximately 3–5 times per week but do not participate in competitions. The control group consisted of healthy women recruited from the general population who did not participate in regular climbing training or competitive sports and engaged in recreational physical activity of no more than 5 h per week. Participants with clinically relevant eating disorder symptoms (EDE-QS > 15) were excluded to reduce potential confounding [21]. Participants accessed the study through a link that directed them to an anonymous electronic survey. Recruitment was performed through climbing-related networks, including national teams and regional performance centers, as well as public advertisement. A flowchart illustrating recruitment, screening, exclusion, and allocation is provided in Supplementary Figure S1. The Ethics Committee of the canton, Bern, Switzerland, reviewed and approved the study protocol (Project-ID: 2021-00221: approval date: 27 April 2021). This study was conducted in full compliance with the ethical principles outlined in the Declaration of Helsinki [22].

### 2.2. Survey Administration and Data Collection

Data was collected using an online questionnaire programmed in REDCap^®^ and was available in German, including standardised and validated instruments: the LEAF-Q (Low Energy Availability in Females Questionnaire), EDE-QS (Eating Disorder Examination Questionnaire Short), FSFI-d (Female Sexual Function Index—German version) [23], and MRS-II (Menopause Rating Scale II). The LEAF-Q is a screening tool used to identify female athletes at risk of low energy availability (LEA) [19]. The LEAF-Q was originally developed for female athletes and was used in the present study as an exploratory screening instrument across the full cohort. It assesses menstrual function, gastrointestinal symptoms, and injury history, with higher scores indicating increased risk. A total LEAF-Q score ≥ 8 indicates an increased risk of LEA [19].

The EDE-QS is a 12-item self-report screening tool assessing core eating-disorder psychopathology over the past seven days [21]. The FSFI-d is a validated, multidimensional self-report questionnaire used to assess female sexual function over the previous four weeks across six domains: desire, arousal, lubrication, orgasm, satisfaction, and pain [24]. The FSFI-d desire domain score was used as a surrogate parameter for libido, whereas the FSFI-d total score was used to assess overall sexual function. This study analysed questionnaire-derived scores rather than clinically diagnosed sexual dysfunction.

The MRS-ll is a validated self-report questionnaire used to assess symptoms related to estrogen deficiency [25]. The questionnaire was distributed via email using a non-personalised access link. It was thematically structured into subsections addressing different aspects of health and lifestyle. These sections included: (1). Personal characteristics and medical history (body mass index, sleep patterns, family history), (2). eating disorder screening (EDE-QS) [21], (3). sexual function (FSFI-d) [24], (4). low energy availability (LEAF-Q) [19], (5). estrogen deficiency symptoms (MRS-II) [25], and (6). dietary habits (including questions on the consumption of foods with low energy density, intermittent fasting, low-carbohydrate diet, and flavored foods).

### 2.3. Statistical Analysis

A priori sample size calculation was performed during study planning. Based on the assumed prevalence of libido disorders of 20% in controls [4] and 45% in elite athletes [5], an effect size of 0.3, a significance level of 5%, and a statistical power of 80%, a total sample size of 180 participants was estimated. Descriptive statistics were calculated for all variables. Categorical variables are presented as frequencies and percentages, while continuous variables are reported as mean, standard deviation (SD), or median with interquartile range (IQR) defined as the range between the 25th and 75th percentiles (Q1, Q3). Group differences between elite climbers, amateur climbers, and the control group were assessed using non-parametric tests due to the relatively small sample size and non-normal distribution of several variables, including the FSFI-d total score. Overall group comparisons between the three groups were performed using the Kruskal–Wallis test. When a significant overall effect was detected, predefined pairwise comparisons were performed using the Mann–Whitney U test (elite versus control, amateur versus control, elite versus amateur). Categorical variables were analysed using the Chi-square test or Fisher’s exact test, where appropriate. Additional adjusted analyses using analysis of covariance (ANCOVA) were performed exploratorily to evaluate potential associations while accounting for relevant covariates. To meet model assumptions, FSFI-d total scores were transformed using a Johnson transformation [26] to approximate a normal distribution prior to analysis. Covariates with a known or plausible association with female sexual function and REDs-related physiology reported in the previous literature (age, MRS-II score, LEAF-Q score, BMI, and subjective sexual function) were evaluated for inclusion in the model [3,5,27]. Following this clinical preselection, factor analysis was used to determine the number of variables retained in the final model. Due to potential interactions and the limited sample size, a reduced model approach was applied. The primary outcome was the FSFI-d total score representing overall sexual function. Secondary outcomes included libido, assessed using the FSFI-d desire domain score, other FSFI-d domain scores, LEAF-Q score, menstrual dysfunction, and estrogen deficiency symptoms. To examine the association between a LEAF-Q score, indicative of low energy availability (≥8), and reduced libido, correlation analysis was performed. Statistical significance was defined as *p* < 0.05. Effect sizes were reported as Cohen’s d for pairwise comparisons and partial η^2^ for ANCOVA models. Analyses were conducted using standard statistical software (SPSS) version 19.0 (SPSS Inc., Chicago, IL, USA) [28].

Group differences between elite climbers, amateur climbers, and the control group were assessed using non-parametric tests due to the relatively small sample size and non-normal distribution of several variables, including the FSFI-d total score.

## 3. Results

### 3.1. Participants Characteristics

A total of 173 female participants aged 16–35 years were included in this study. Of these, 31 (17.9%) were assigned to the group of elite climbers, 55 (31.8%) to amateur climbers, and 87 (50.3%) to the control group. Detailed baseline participant characteristics of the study population are presented in Table 1. Significant differences between groups were observed for age, body weight, BMI, and training volume. Elite climbers were significantly younger and had lower body weight and BMI compared with amateur climbers and the control group (*p* < 0.001). Training hours per week differed significantly between groups (*p* < 0.001), consistent with the predefined group classification.

### 3.2. Sexual Function (FSFI-d Total Score and Subscores, Sexual Activity)

**Figure 1:**
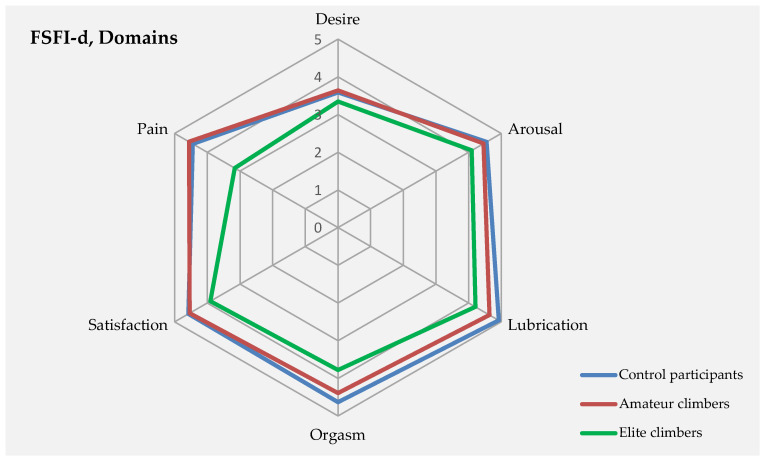
FSFI-d domain scores by study group. Radar plot of mean FSFI-d domain scores (desire, arousal, lubrication, orgasm, satisfaction, and pain) by study group (controls, amateur climbers, and elite climbers). Higher scores indicate better sexual function.

The FSFI-d total score, representing overall sexual function, did not differ significantly between the control group, the amateur climbers, and the elite climbers in the primary unadjusted analysis (*p* = 0.518). Median FSFI-d scores were 28.3 (IQR 14.3–32.1) in elite climbers, 30.0 (IQR 23.7–31.8) in amateur climbers, and 27.3 (IQR 23.4–30.8) in the control group (Table 2). No statistically significant differences were observed in any of the FSFI-d subdomains (desire, arousal, lubrication, orgasm, satisfaction, pain) between the three study groups (Table 2), although a consistent pattern of lower domain scores in elite climbers is illustrated in Figure 1. Additional graphical presentation, including variability measures, is provided in Supplementary Figure S2. In particular, the desire domain (libido) did not differ significantly between the groups (*p* = 0.610) (Table 2), and pairwise comparison between elite climbers and controls also showed no significant difference in the FSFI-d subdomain libido (*p* = 0.430). Among the 134 participants reporting sexual activity within the previous four weeks (134 of 173 77.5%), FSFI-d total scores did not differ significantly between groups in the overall comparison (*p* = 0.123). However, pairwise comparison within this subgroup revealed a statistically significant difference between elite climbers and the control group (*p* = 0.044), whereas no differences were observed between amateur climbers and the control group (*p* = 0.494) or between amateur climbers and elite climbers (*p* = 0.125). In adjusted analyses using ANCOVA on transformed data, a significant group effect was observed between elite climbers and the control group (F = 14.692; *p* < 0.001), indicating lower FSFI-d total scores in elite climbers after accounting for the covariates age, MRS-II score, LEAF-Q score, BMI, and subjective sexual satisfaction (Table 3). The covariate MRS-ll score (*p* = 0.003) and subjective sexual satisfaction (*p* < 0.001) showed a statistically significant association with the FSFI-d total score, whereas LEAF-Q score, age, and BMI were not significant predictors in the ANCOVA model.

There were statistically significant differences in sexual activity between the study groups. A higher proportion of elite climbers reported never having had sexual intercourse compared with amateur climbers and the control group (elite climbers: 22.6%, amateur climbers: 5.5%, control group: 1.1%; *p* < 0.001). Despite this, elite climbers reported significantly higher satisfaction with their sexual life than the control group (mean elite group: 4.07 ± 0.91; mean control group: 3.43, ± 1.06, *p* = 0.002). Age at first intercourse did not differ significantly between the study groups (overall Mean 17.4 SD ± 2.0 years; *p* = 0.845).

### 3.3. Low Energy Availability (LEAF-Q) and Menstrual Dysfunction

The prevalence of high risk for low energy availability (LEA; LEAF-Q score ≥ 8) did not differ between elite climbers, amateur climbers, and controls. Mean LEAF-Q scores were 7.3 ± 4.7 in the elite climbers, and 7.0 ± 5.6 in the amateur climbers compared to 5.6 ± 3.8 in the control group (*p* = 0.059). No statistically significant differences were observed in self-reported menstruation function, defined as reporting a regular menstrual cycle (elite climbers: 74.2%, amateur climbers: 87.3%, control group: 86.2%; *p* = 0.071). Age at menarche, grouped into age classes, differed significantly between groups (*p* = 0.008), with elite climbers reporting a later age at menarche (≥15 years: 29.0%) compared with amateur climbers (25.5%) and controls (17.2%). Most participants in all groups reported menstruation within the previous four weeks, consistent with eumenorrhea (control: 93.3%, amateur: 97.9%, elite: 87.0%). Intervals of more than three months since the last menstruation, consistent with secondary amenorrhea [30], were rare overall (elite climbers: 4.3%, amateur climbers: 0%, control group: 4.0%) and did not suggest a higher prevalence among climbers.

### 3.4. Estrogen Deficiency Symptoms (MRS-II)

The MRS-II total score did not differ significantly among the three study groups (elite climbers: 7.65 ± 6.50, amateur climbers: 7.18 ± 4.66, control group: 6.48 ± 5.21; *p* = 0.398). Mean scores tended to be higher in both groups of climbers (elite and amateur) compared with controls, although this did not reach statistical significance. However, higher MRS-II scores were significantly associated with lower FSFI-d scores (correlation r = −0.299; *p* < 0.001). The MRS-II total score, as well as the following individual items, correlated significantly with the FSFI-d total score: hot flushes (r = −0.225; *p* =0.003), sleep problems (−0.266; *p* < 0.001), depressive mood (r = 0.226; *p* = 0.003), irritability (r = −0.159; *p* = 0.038), physical and mental exhaustion (r = −0.188; *p* = 0.013), sexual problems r = −339; *p* < 0.001), and vaginal dryness (r = −0.183: *p* = 0.016).

### 3.5. Eating Disorder (EDE-QS)/Eating Habits

Among the participants included in the study, overall EDE-QS scores did not differ significantly between the study groups. Nevertheless, the analysis of individual questionnaire items revealed statistically significant differences for selected EDE-QS items. For example, item 4 (‘How many of the past 7 days has thinking about your weight or shape made it difficult to concentrate on things you are interested in?’) differed significantly between groups (mean score elite climbers = 0.48, amateur climbers = 0.31, control group = 0.24; *p* = 0.042). EDE-QS items are scored on a 4-point scale ranging from 0 (no days) to 3 (6–7 days), indicating frequency within the past week. Thus, the observed mean values across all groups correspond to very low symptom frequency for eating disorders, despite statistical significance. The EDE-QS scores were significantly associated with FSFI-d total scores (r = −0.189; *p* = 0.013), indicating a possible relationship between eating related-psychopathology and sexual function.

## 4. Discussion

### 4.1. Libido in Female Athletes

Libido refers to an individual’s sexual desire and reflects the motivation to engage in sexual activity [31]. It arises from the interaction of hormonal, psychological, neurological, and relational factors, and is a key determinant of overall sexual well-being. In our cross-sectional cohort, no statistically significant differences in sexual desire were observed between female climbers (elite and amateur) and women in the control group. This suggests that libido may remain relatively stable despite potential variations in training exposure or REDs-related risk factors. However, these findings should be interpreted cautiously, as the relatively small elite climber subgroup may have limited statistical power to detect subtle group differences, particularly in domain-specific analyses.

In men, the 2023 International Olympic Committee (IOC) consensus update describes low libido and reduced morning erections as emerging physiological indicators of REDs, reflecting the impact of low energy availability on hypothalamic–pituitary–gonadal axis function [6]. In women, however, diagnostic emphasis is placed primarily on impaired reproductive function, such as menstrual disturbances and hormonal alterations, while reduced libido is considered a possible but less prominent feature [32]. Loss of libido represents a relatively specific clinical marker of functional hypogonadism in men, often paralleling reductions in circulating total testosterone concentrations [6,33]. Evidence from male athletic populations illustrates a strong association between energy deficiency and libido changes. In endurance runners, marathon training has been associated with approximately 20% lower libido, with cumulative training years and high-intensity load identified as key predictors, likely mediated by reduced total testosterone availability [16,34]. Experimental data from prolonged athletic competition also demonstrate transient reductions in testosterone and sexual motivation during periods of peak energetic stress [35]. Within the framework of life history theory, such changes can be interpreted as adaptive reallocations of energy away from reproductive function toward survival and performance-related processes [36,37]. In contrast, libido and sexual function in women seem to be highly multifactorial and not determined by sex steroid hormones alone [38]. Additionally, from a hormonal perspective, female libido and sexual function are influenced by both estrogens and androgens [39]. Estrogen is primarily regulated by the hypothalamic–pituitary–gonadal-axis and may be suppressed in the context of REDs [6]. Androgens are primarily produced both in the ovaries and the adrenal glands [40], unlike men, where circulating testosterone is produced predominantly by the testes. Consequently, circulating androgen availability in women reflects the integrated activity of several endocrine pathways rather than ovarian function alone [41]. Adrenal androgen production and peripheral steroid conversion may partly compensate for hypothalamic suppression in women. As a result, female libido may not be solely determined by ovarian endocrine function and likely reflects the integrated activity of multiple endocrine pathways. However, this mechanism was not directly assessed in the present study. Consequently, loss in sexual desire or libido loss appears to be a non-specific and potentially insensitive indicator of REDs in female athletes, which is consistent with the absence of group differences in our study cohort, reflecting the complex and multifactorial regulation of female sexual function. These data highlight further important sex-specific differences in the interpretation of libido within the REDs framework.

### 4.2. Sexual Function in Female Athletes

Sexual function is a multidimensional construct encompassing desire, arousal, orgasm, and pain, shaped by biological, psychological, and interpersonal factors. Female sexual dysfunction (FSD) involves impairments across these domains, accompanied by distress [42]. Prevalence estimates vary due to inconsistent definitions and diagnostic criteria [30]. Population-based studies suggest that up to 40–50% of women report some degree of sexual dysfunction, whereas clinically relevant dysfunction affects approximately 12–19% of women of reproductive age [29]. In athletes, factors associated with low energy availability (LEA)—including high training load, fatigue, sleep disturbances, and hypoestrogenic states—may adversely affect specific aspects of sexual function. In our online cross-sectional survey, no statistically significant differences in FSFI-d full-scale scores were observed across all study groups in unadjusted analyses, likely reflecting the relatively healthy study population and substantial interindividual variability. In addition, the relatively small number of elite female climbers, which partly reflects the limited population of elite climbers in German-speaking countries despite multinational recruitment, may have reduced statistical power, particularly for subgroup and domain-specific analyses.

However, after adjustment for MRS-II total score and sexual satisfaction, pairwise comparison revealed that elite climbers demonstrated significantly lower FSFI-d total scores compared with controls. The discrepancy between unadjusted and adjusted analyses highlights the importance of potential confounders when assessing sexual function in female athletes. In particular, estrogen-related symptoms and subjective sexual satisfaction appear to influence FSFI-d scores.

Whether this observation reflects a true difference between elite climbers and controls should be examined in larger future studies. Interestingly, subjective satisfaction with sexual life was higher in elite climbers despite lower FSFI-d scores. This discrepancy is clinically relevant, as female sexual dysfunction requires not only symptoms but also associated subjective distress [42]. Therefore, lower FSFI-d scores alone do not necessarily indicate clinically relevant sexual dysfunction. This apparent dissociation may have several explanations. Elite athletes may prioritise performance over certain aspects of sexual function. In addition, psychosocial factors such as expectations, coping strategies, and body image may influence subjective sexual satisfaction.

Comparisons with existing athletic literature highlights the heterogeneity of sexual health outcomes across sports. A study of master athletes suggests that sustained moderate-to-vigorous exercise may preserve or even enhance sexual desire (male and female participants) and satisfaction (female participants), challenging the assumption that high training loads universally impair sexuality [5]. Physiological studies provide further context, showing that elite female athletes have been shown to exhibit increased clitoral blood flow and improved arousal-related FSFI domains without changes in sexual desire, supporting the concept that exercise may preferentially influence vascular or physiological components of sexual response rather than libido itself [43]. Nevertheless, these studies used different methodologies and assessed different athletic populations, limiting direct comparability with the present cohort. Conversely, participation in lean sports has been associated with a higher prevalence of eating disorders and amenorrhea compared with non-lean sports [44,45]. Both eating disorders and associated amenorrhea are well-recognised conditions associated with impaired sexual function and diminished libido [46,47,48]. Pelvic floor and urogenital symptoms represent another important dimension of sexual health in athletes. High prevalence rates of dyspareunia, urinary incontinence, pelvic pain, and genital discomfort have been reported across multiple athletic disciplines (various disciplines of athletics, dancing, soccer, gymnastics, cycling), often independent of libido measures [49,50,51]. In cycling, symptoms are predominantly attributed to mechanical factors, particularly perineal pressure. Cycling studies demonstrate strong associations between genital numbness and reduced arousal, lubrication, and satisfaction, whereas desire remains less consistently affected [52,53]. In contrast, climbing is not associated with sustained perineal loading. Therefore, systemic factors such as energy availability and endocrine regulation may be more relevant determinants of sexual function in this discipline.

### 4.3. Low Energy Availability, Menstrual Dysfunction

No significant differences were observed in overall LEAF-Q full-scale scores or in the self-reported presence of a “normal” menstrual cycle between the study groups, suggesting no overt signs of low energy availability at the group level. This finding may partly reflect the exclusion of participants with eating disorder symptoms, potentially reducing the representation of individuals at higher risk for REDs. A more detailed analysis revealed subtle group differences: while most participants reported recent menstruation consistent with eumenorrhea, the proportion was slightly lower in elite climbers, who also reported a later age at menarche. Prolonged intervals exceeding three months, consistent with secondary amenorrhea, were rare and did not differ between groups. These relatively low rates of menstrual disturbances are lower than previously reported in climbing populations, where 15.8% of female sport climbers reported current amenorrhea and 12.3% reported irregular menstrual cycles in the 2022 World Climbing survey [54]. The comparatively low prevalence of menstrual disturbances observed in our cohort compared with previous climbing studies may partly reflect methodological differences, including the exclusion of participants with elevated EDE-QS scores. Notably our questionnaire relies on self-reported menstrual characteristics, including the participant’s perception of having a “normal” menstrual cycle. Some athletes reported a normal cycle despite prolonged intervals exceeding five months, indicating discrepancies between perceived and physiological menstrual health. This highlights a well-recognised challenge in female athlete health research, as menstrual health literacy remains limited among female athletes and their support networks (e.g., coaches) [55].

### 4.4. Estrogen Deficiency Symptoms

No significant differences were observed in MRS-II scores between the study groups, suggesting a generally low symptom burden. However, hypoestrogenic states in premenopausal athletes may manifest through subtle changes rather than the classic vasomotor or psychological symptoms captured by menopause-focused instruments. As sex hormones were not measured in the present study, conclusions regarding estrogen status cannot be drawn directly from our data. To date, no validated questionnaire exists to specifically assess hypoestrogenism or REDs-related endocrine dysfunction in premenopausal athletes.

### 4.5. Eating Disorders

Climbing is widely regarded as a lean sport in which performance is often associated with low body mass, and an elevated risk of disordered eating behavior has been reported. In a large international cohort of approximately 400 sport climbers, the overall prevalence of eating disorders was 12.5%, with substantially higher rates of up to 43% among female elite climbers performing at advanced levels (≥8a+) [56]. Given this background, eating-related psychopathology represents an important potential modifier of both endocrine function and sexual health outcomes in climbing populations. Evidence from clinical populations indicates that sexual dysfunction is highly prevalent across eating disorder subtypes, with reduced sexual desire reported by up to 66.9% of affected women [46]. However, studies systematically examining sexual function specifically in female athletes with eating disorders remain scarce. In the present study, participants with diagnosed or suspected eating disorders were excluded based on predefined exclusion criteria. All participants were screened using the EDE-QS, and individuals scoring above the established cut-off (>15 points) [21] were automatically excluded from further analysis, resulting in a comparatively healthy study cohort.

While this approach strengthened internal validity and reduced confounding variables from severe psychopathology, it may also have introduced a healthy-samples bias, reducing variability in relevant metabolic and psychological factors and potentially attenuating associations between energy availability and sexual function. Consistent with this, no significant differences in EDE-QS scores were observed between groups, and overall symptom levels were low.

## 5. Limitations

Several limitations should be considered when interpreting the findings of this study. First, the relatively small sample size may have reduced statistical power, particularly for subgroup and domain-specific analyses. Second, participants with clinically relevant eating disorders were excluded to minimise confounders, resulting in a comparatively healthy study population and a potential healthy-sample bias. There was also a significant age difference between elite climbers, amateur climbers, and controls, which may have influenced sexual function. Several other relevant confounders were not systematically assessed, including contraception, partnership status, sexual orientation, sexual experience, body image-related factors, and hormonal parameters, all of which may influence sexual function.

## 6. Conclusions

In this cross-sectional cohort study of female elite and amateur climbers, overall sexual function and libido did not differ significantly between climbers (amateur and elite) and controls. However, elite climbers demonstrated lower FSFI-d total scores in pairwise comparisons after adjusting for covariates like MRS-II total score and sexual satisfaction, indicating potential differences in sexual function associated with elite competitive sport. Despite these differences, subjective sexual satisfaction was higher in elite athletes, highlighting the multidimensional nature of sexual health in physically active women. From a clinical perspective, these findings highlight the multidimensional nature of sexual health and suggest that libido alone may not adequately reflect underlying physiological alterations, including those related to REDs.

## Figures and Tables

**Table 1:** Participant characteristics by study group.

	Control (n = 87) Mean ± SD Median (1st; 3rd Quartile)	Elite (n = 31) Mean ± SD Median (1st; 3rd Quartile)	Amateur (n = 55) Mean ± SD Median (1st; 3rd Quartile)	*p*-Value (E vs. C/A vs. C/E vs. A)
Age (years)	28.2 ± 4.5 28.0 (24.0; 31.0)	22.1 ± 4.3 22.0 (19.0; 24.0)	28.4 ± 4.4 28.0 (25.0; 33.0)	<0.001 (<0.001/0.851/<0.001)
Height (cm)	167.2 ± 6.4 168.0 (163.0; 172.0)	165.4 ± 5.0 165.0 (163.0; 169.0)	166.5 ± 6.6 166.0 (162.0; 170.0)	0.426
Weight (kg)	62.6 ± 11.5 60.0 (56.0; 65.0)	55.7 ± 5.5 55.0 (51.0; 59.0)	58.3 ± 6.1 59.0 (54.0; 62.0)	0.001 (<0.001/0.035/0.035)
BMI (kg/m^2^)	22.4 ± 3.7 21.83 (20.2; 23.5)	20.3 ± 1.6 20.0 (19.2; 21.5)	21.0 ± 1.9 20.7 (19.6; 22.32)	<0.001 (<0.001/0.019/0.070)
Training hours (h/week)	3.8 ± 2.0 4.0 (2.0; 5.0)	18.4 ± 5.9 18.0 (15.0; 20.0)	11.7 ± 7.4 10.0 (7.0; 14.0)	<0.001 (each < 0.001)

Abbreviations: BMI = body mass index.

**Table 2:** FSFI-d total and domain scores by study group.

FSFI-d Domain	Control (n = 87) Mean ± SD, Median (1st; 3rd Quartile)	Elite (n = 31) Mean ± SD, Median (1st; 3rd Quartile)	Amateur (n = 55) Mean ± SD, Median (1st; 3rd Quartile)	*p*-Value Cohen’s d (C vs. E/C vs. A/A vs. E)
**Total score**	26.72 ± 6.74 28.30 (23.40, 30.80)	22.51 ± 11.77 28.30 (14.30, 32.10)	26.24 ± 8.80 30.00 (23.70, 31.80)	0.518 0.50/0.06/0.37
**Desire**	3.59 ± 1.09 3.60 (3.00, 4.20)	3.35 ± 1.32 3.60 (2.10, 4.80)	3.64 ± 1.11 3.60 (3.00, 4.20)	0.610 0.21/0.05/0.24
**Arousal**	4.55 ± 1.38 4.80 (3.90, 5.40)	4.10 ± 2.18 5.10 (3.00, 5.70)	4.46 ± 1.68 5.10 (3.90, 5.40)	0.956 0.28/0.06/0.19
**Lubrication**	4.93 ± 1.44 5.40 (4.50, 6.00)	4.21 ± 2.42 5.70 (3.60, 6.00)	4.64 ± 1.75 5.40 (4.20, 6.00)	0.757 0.41/0.19/0.21
**Orgasm**	4.63 ± 1.45 5.20 (4.00, 6.00)	3.78 ± 2.27 4.80 (2.40, 5.60)	4.39 ± 1.74 4.80 (4.00, 5.60)	0.324 0.50/0.15/0.31
**Satisfaction**	4.58 ± 1.67 5.20 (4.00, 6.00)	3.91 ± 2.47 5.20 (1.20, 6.00)	4.54 ± 1.92 5.20 (4.40, 6.00)	0.847 0.43/0.02/0.30
**Pain**	4.44 ± 2.21 5.20 (4.00, 6.00)	3.16 ± 2.73 4.00 (0.00, 6.00)	4.56 ± 2.01 5.20 (4.40, 6.00)	0.107 0.54/0.06/0.61

Abbreviations: SD = standard deviation; values are presented as mean ± SD and median (IQR). *p*-values refer to overall group comparisons. Cohen’s d values are reported for pairwise group comparisons. An FSFI-d total score ≤ 26.55 has been suggested as indicative of female sexual dysfunction [29].

**Table 3:** ANCOVA results for FSFI-d total score comparing elite climbers and controls.

Variable	F-Value	*p*-Value	η^2^
**Group effect unadjusted (elite vs. control)**	2.398	0.124	0.020
**Age**	0.312	0.578	0.003
**MRS-II score**	8.991	0.003	0.073
**LEAF-Q score**	0.105	0.747	0.001
**BMI**	0.052	0.821	<0.001
**Subjective sexual satisfaction**	87.610	<0.001	0.434
**Group effect adjusted by MRS-II score and subjective sexual satisfaction (ANCOVA model, elite vs. control)**	14.692	<0.001	0.116

ANCOVA results adjusted for age, MRS-II score, LEAF-Q score, BMI, and subjective sexual satisfaction. FSFI-d total score was transformed to meet model assumptions.

## Data Availability

The raw data supporting the conclusions of this article will be made available by the authors, without undue reservation.

## Supplementary Materials

The following supporting information can be downloaded at: https://www.mdpi.com/article/10.3390/sports14060242/s1, Supplementary Figure S1: Flowchart of the study sample with participant recruitment and allocation; Supplementary Figure S2: Mean FSFI-d domain scores across study groups.
