# Supplementary material for: Sexual Function and Libido Loss in Female Climbers—A Cross-Sectional Study
Source: Sports (Basel). 2026 Jun 11;14(6):242. doi: 10.3390/sports14060242 (PMC13306252; doi:10.3390/sports14060242)
Supplement: Supplementary file 1 [file sports-14-00242-s001.zip › sports-4298772-supplementary Figure S1.pdf]

Supplementary Figure S1

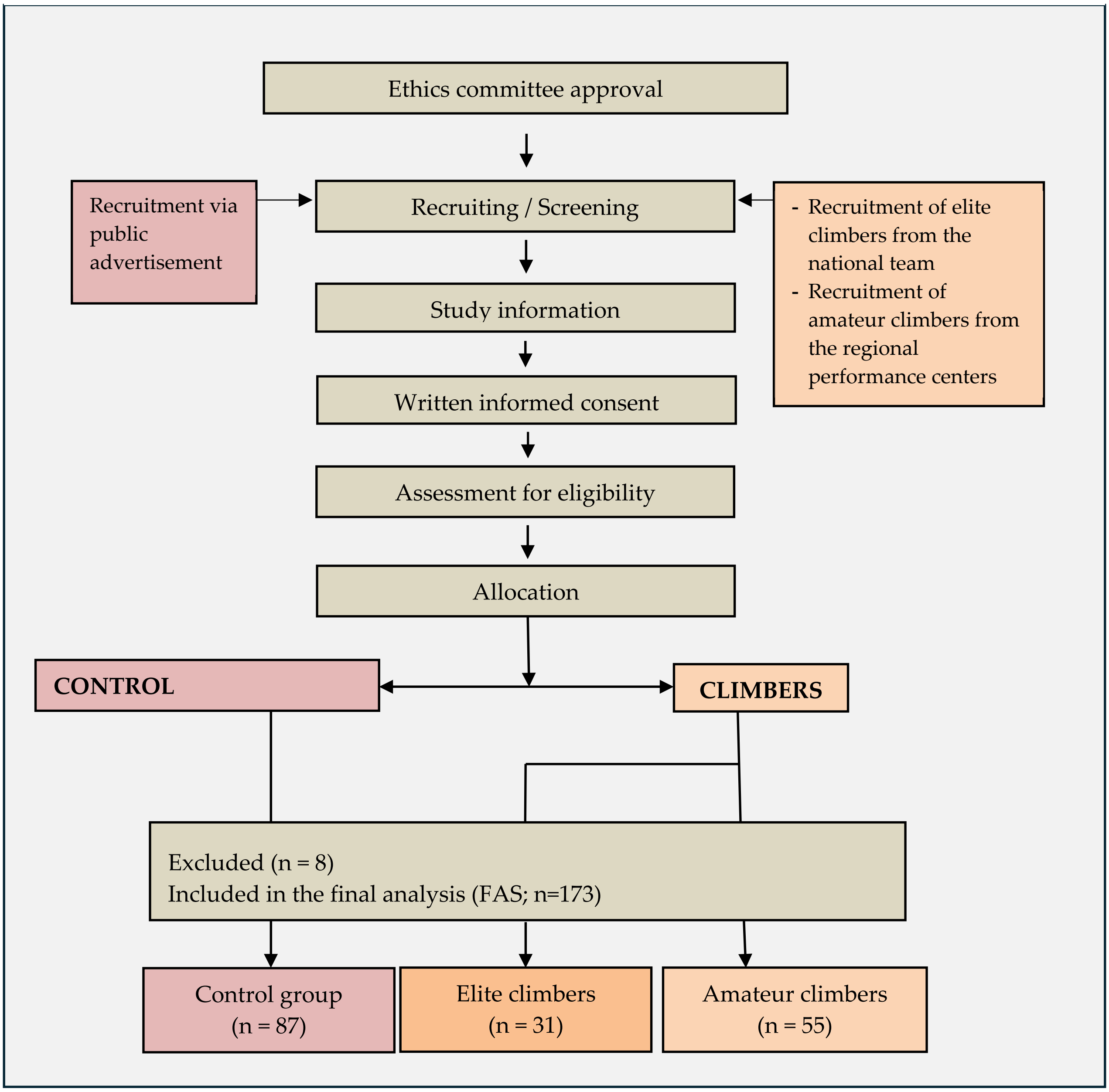

Flowchart of the study sample with participant recruitment and allocation
